# Coenzyme Q_0_ Inhibits NLRP3 Inflammasome Activation through Mitophagy Induction in LPS/ATP-Stimulated Macrophages

**DOI:** 10.1155/2022/4266214

**Published:** 2022-01-07

**Authors:** You-Cheng Hseu, Yu-Fang Tseng, Sudhir Pandey, Sirjana Shrestha, Kai-Yuan Lin, Cheng-Wen Lin, Chuan-Chen Lee, Sheng-Teng Huang, Hsin-Ling Yang

**Affiliations:** ^1^Department of Cosmeceutics, College of Pharmacy, China Medical University, Taichung 40402, Taiwan; ^2^Department of Health and Nutrition Biotechnology, Asia University, Taichung 41354, Taiwan; ^3^Chinese Medicine Research Center, China Medical University, Taichung 40402, Taiwan; ^4^Research Center of Chinese Herbal Medicine, China Medical University, Taichung 40402, Taiwan; ^5^Institute of Nutrition, College of Health Care, China Medical University, Taichung 40402, Taiwan; ^6^Department of Medical Research, Chi Mei Medical Center, Tainan 71004, Taiwan; ^7^Department of Biotechnology, Chia Nan University of Pharmacy and Science, Tainan 71710, Taiwan; ^8^Department of Medical Laboratory Science and Biotechnology, China Medical University, Taichung 40402, Taiwan; ^9^Department of Chinese Medicine, China Medical University Hospital, Taichung 40402, Taiwan

## Abstract

Coenzyme Q (CoQ) analogs with a variable number of isoprenoid units have exhibited as anti-inflammatory as well as antioxidant molecules. Using novel quinone derivative CoQ_0_ (2,3-dimethoxy-5-methyl-1,4-benzoquinone, zero side chain isoprenoid), we studied its molecular activities against LPS/ATP-induced inflammation and redox imbalance in murine RAW264.7 macrophages. CoQ_0_'s non- or subcytotoxic concentration suppressed the NLRP3 inflammasome and procaspase-1 activation, followed by downregulation of IL1*β* expression in LPS/ATP-stimulated RAW264.7 macrophages. Similarly, treatment of CoQ_0_ led to LC3-I/II accumulation and p62/SQSTM1 activation. An increase in the Beclin-1/Bcl-2 ratio and a decrease in the expression of phosphorylated PI3K/AKT, p70 S6 kinase, and mTOR showed that autophagy was activated. Besides, CoQ_0_ increased Parkin protein to recruit damaged mitochondria and induced mitophagy in LPS/ATP-stimulated RAW264.7 macrophages. CoQ_0_ inhibited LPS/ATP-stimulated ROS generation in RAW264.7 macrophages. Notably, when LPS/ATP-stimulated RAW264.7 macrophages were treated with CoQ_0_, Mito-TEMPO (a mitochondrial ROS inhibitor), or *N*-acetylcysteine (NAC, a ROS inhibitor), there was a significant reduction of LPS/ATP-stimulated NLRP3 inflammasome activation and IL1*β* expression. Interestingly, treatment with CoQ_0_ or Mito-TEMPO, but not NAC, significantly increased LPS/ATP-induced LC3-II accumulation indicating that mitophagy plays a key role in the regulation of CoQ_0_-inhibited NLRP3 inflammasome activation. Nrf2 knockdown significantly decreased IL1*β* expression in LPS/ATP-stimulated RAW264.7 macrophages suggesting that CoQ_0_ inhibited ROS-mediated NLRP3 inflammasome activation and IL1*β* expression was suppressed due to the Nrf2 activation. Hence, this study showed that CoQ_0_ might be a promising candidate for the therapeutics of inflammatory disorders due to its effective anti-inflammatory as well as antioxidant properties.

## 1. Introduction

NLRP3 is a commonly studied inflammasome complex that is named after the NLRP3 protein of the Nod-like receptor (NLR) family [1]. NLRP3 is a cytosolic protein of 115 kDa which is expressed in monocytes, neutrophils, dendritic cells, epithelial cells, and lymphocytes [2]. The NLRP3 inflammasome activation is a tightly regulated process that needs priming as well as activation signals [3]. The NLRP3 inflammasome activation is associated with age-related diseases [4, 5] as well as different types of cancer [6]. A variety of targets can be applied for its repression by taking benefits of the complex NLRP3 inflammasome signaling cascades, such as inhibition of NLRP3 inflammasome activation, inhibition of caspase-1 activation, suppression of upstream signals, and neutralization of inflammatory cytokines secreted by NLRP3 inflammasome [7].

Autophagy is an evolutionarily conserved catabolic mechanism that involves the generation of vesicles known as autophagosomes which engulf macromolecules and organelles of the cell and fuses with lysosomes for their breakdown [8]. Autophagic cell death is often referred to as programmed cell death of Type II which differs from Type I cell death including apoptosis and necrosis-like cell death mechanism [9]. One of the cases of autophagic cell death is due to the production of an increased amount of reactive oxygen species (ROS) that results from autophagic degradation of catalase [10]. Defects in autophagy play a vital role in the pathogenesis of several diseases including aging [11] and cancer [12]. Recent evidence revealed that autophagy has an important function in the development as well as the pathogenesis of inflammation and immunity response [13]. In inflammation, autophagy plays a critical role by affecting the development, survival, and homeostasis of inflammatory cells such as neutrophils, macrophages, and lymphocytes [14]. Removal of NLRP3 inflammasome and cytokine components by autophagy can suppress the activation of the inflammasome and inflammatory response. Similarly, pathways related to inflammasome can control autophagy for the balance between host defense inflammatory response and prevention of excessive and harmful inflammation [15]. The NF-E2-related factor 2 (Nrf2) transcription factor is a key mediator of the expression of cytoprotective genes which are activated during stress conditions due to the generation of ROS [16]. The increasing number of evidence supports that there is a crosstalk between the Nrf2 and inflammasome pathways at various levels. Inflammasomes and thus inflammation are inhibited by Nrf2-activating compounds [17].

Mitophagy is the process of removing damaged mitochondria through autophagy [18]. The injured mitochondria are engulfed in the autophagosomal membrane to turn into the autophagosome and then fuse with lysosomes, which are later degraded by hydroxylases [19]. Parkin and PTEN-induced kinase 1 (PINK1) play an important role in mitochondrial homeostasis and mitophagy [20]. Parkin is an E3 ligase that ubiquitinates outer mitochondrial membrane proteins, allowing the autophagic removal of the damaged organelles [21]. Parkin can regulate autophagy of damaged mitochondria, and its overexpression leads to induction of complete removal of mitochondria from cells by the process of mitophagy where membrane potential of mitochondria is lost [22]. Since Parkin selectively binds only to damaged mitochondria, there are speculations that it can mediate the mitophagy quality control pathway [23]. Disruption in Parkin-PINK1 signaling leads to impaired mitophagy [24]. Hence, gaining insight into the Parkin/PINK1/mitophagy pathway might help to understand the pathogenic signaling pathways.

Coenzyme (CoQ) is a ubiquinone analog available in all cells and membranes, and being a member of the mitochondrial respiratory chain, it functions in cellular metabolism [25]. CoQ_0_ consists of a benzoquinone ring conjugated to an isoprenoid chain. Depending upon the number of isoprenoid side chains, CoQ varies from CoQ_0_ to CoQ_10_ [26]. The antioxidant property of any compound is represented when it prevents oxidative stress-induced cell death [27]. Various analogs of CoQ_0_ have shown antioxidant or proantioxidant properties [28, 29]. CoQ_0_ is a coenzyme and redox-active compound without an isoprenoid side chain occurring mostly within mitochondria that suppresses the activity of complex 1 of the mitochondrial respiratory chain and prevents the opening of the mitochondrial permeability transition pore [30]. Recently, many studies have reported that CoQ_0_ has therapeutic effects on inflammation, metabolic disorders, and cancer [31–34]. CoQ_0_ improved atopic dermatitis-like wounds by reducing IL1*β*, IL4, IL6, IL10, and interferon (IFN) *γ* and by infiltrating neutrophils in the lesional skin [35]. ROS production by external stimuli along with lipopolysaccharide (LPS) promotes inflammatory response in cultured macrophages [36]. However, the main pharmacological efficacy against inflammation and redox imbalance of CoQ_0_ molecule has not been thoroughly examined, and the signaling pathways regulated by it remain largely unknown. Therefore, we examined if CoQ_0_ treatment could reduce the LPS-induced inflammatory response as well as redox imbalance in LPS- and ATP-induced RAW264.7 macrophages.

Lipopolysaccharide (LPS) is a powerful monocyte and macrophage activator and induces the secretion of numerous proinflammatory molecules, cytokines, nitric oxide (NO), tumor necrosis factor-alpha, and interleukins including IL1 or IL6 [37], which are responsible for the development and progression of inflammatory disease and cancer. One of the most characterized pathogen-associated molecular patterns (PAMPs) is LPS, which is the major constituent of the external membrane of Gram-negative bacteria [38]. Macrophage activation by LPS has been broadly studied to explore the inflammatory phenomenon in both cell culture and animal models [39]. During inflammation and infection, LPS can activate various signals within macrophages [40]. LPS activation of macrophages can cause an increase in oxygen absorption, leading to a range of reactive oxygen species (ROS), which are the key factors that drive oxidative stress-stimulated inflammation in immune cells [41]. In the extracellular environment, ATP is actively released in response to tissue harm and cellular stress [42]. ATP, generally secreted from dying and stressed cells, is used as a damage-associated molecular pattern to activate NLRP3 inflammation [43]. Nrf2 belongs to the base-leucine zipper (bZIP) which is the family of transcriptional activator proteins and triggered by endogenous oxidative stress [44]. It mediates cellular antioxidant responses by controlling the expression of genes that encode detoxifying enzymes and antioxidants [45]. Several pieces of evidence suggest that Nrf2 plays a crucial role in guarding macrophages against LPS-stimulated inflammation [46].

Previously, our *in vitro* and *in vivo* studies showed that CoQ_0_ (2,3-dimethoxy-5-methyl-1,4-benzoquinone), a novel quinone derivative, regulated NF*κ*B/AP-1 activation and enhanced Nrf2 stabilization in attenuation of LPS-induced inflammation and redox imbalance [29]. The noncytotoxic concentrations of CoQ_0_ (2.5-10 *μ*M) inhibited iNOS/COX-2 protein expression in LPS-stimulated macrophages, resulting in lower NO, PGE2, INF, and IL1 secretions. Moreover, in LPS-stimulated macrophages, CoQ_0_ induced the expression of HO-1 and NQO-1 genes by increasing Nrf2 nuclear translocation and Nrf2/ARE signaling [29]. However, studies regarding the effect of CoQ_0_ against LPS/ATP-induced inflammation and redox imbalance using murine macrophages have still not been carried out. In this study, we explored CoQ_0_ molecular activities against LPS/ATP-induced inflammation as well as redox imbalance in murine RAW264.7 macrophages. These results proposed that CoQ_0_ negatively regulates activation of macrophage by inducing autophagy and activation of Nrf2 and hindering a positive feedback loop of NLRP3 inflammasome pathways and may be a potential therapeutic target for inflammatory diseases because of its potent anti-inflammatory and antioxidant properties.

## 2. Materials and Methods

### 2.1. Chemicals and Reagents

Dulbecco's modified Eagle's medium (DMEM), fetal bovine serum (FBS), glutamine, penicillin-streptomycin, ATP, and Mito-TEMPO were bought from the Invitrogen/GIBCO BRL (Grand Island, NY, USA). LPS (from *Escherichia coli* 055: B5), Coenzyme Q_0_ (2,3-dimethoxy-5-methyl-1,4-benzoquinone), 2′,7′-dihydrofluorescein-diacetate (DCFH_2_-DA), 3-(4,5-dimethylthiazol-2-yl)-2,5-diphenyltetrazolium bromide (MTT), *N*-acetylcysteine (NAC), and cycloheximide were bought from Sigma-Aldrich (St. Louis, MO, USA). Antibodies against NLRP3 were obtained from Biorbyt (Cambridge, UK). Antibodies against IL1*β* and Parkin were acquired from Abcam (Cambridge, UK). Antibodies against Nrf2, p70 S6 kinase, p-p70 S6 kinase, procaspase-1, GAPDH, and *β*-actin were procured from Santa Cruz (Heidelberg, Germany). Antibodies against p62, Beclin-1, Bax, mTOR, AKT, PI3K, p-mTOR, p-AKT, p-PI3K, and histone H3 were brought from Cell Signaling Technology Inc. (Danvers, MA). 4′,6-Diamidino-2-phenylindole dihydrochloride (DAPI) was purchased from Calbiochem (La Jolla, CA, USA). Antibodies against PINK1 were purchased from Genetic Technology Inc. (Miami, FL, USA). The rest of the chemicals were of the highest commercially available grade and supplied by either Sigma (St. Louis, MO, USA) or Merck (Darmstadt, Germany).

### 2.2. Cell Culture and Sample Treatment

RAW264.7 cells which were derived from murine macrophage were purchased from American Type Culture Collection (ATCC, Rockville, MD, USA). The cells were then cultured in DMEM that contained 2 mM glutamine, 1% penicillin-streptomycin, and 10% FBS at 37°C in a humidified atmosphere of 5% CO_2_. After incubating with CoQ_0_ for 1 h, the supernatant was removed and the cells were washed with phosphate buffer saline (PBS). After washing with PBS, the culture medium was displaced with a new medium with or without LPS (1 *μ*g/mL) dissolved in PBS having pH 7.2 and ATP (5 mM) for the designated time.

### 2.3. MTT Assay

1 × 10^5^ RAW264.7 cells/well were cultured on a 24-well plate till confluence and then incubated with 2.5-20 *μ*M CoQ_0_ for 24 h. To monitor cell viability, after treatment with CoQ_0_, the cells were further incubated with 400 *μ*L of 0.5 mg/mL of MTT along with medium for 1 h. Post incubation, the supernatant was discarded, and thus, formed formazan crystals were dissolved in 400 *μ*L of dimethyl sulfoxide (DMSO). The absorbance was estimated using an enzyme-linked immunosorbent assay (ELISA) microplate reader (BioTek Instruments, Winooski, VT, USA) at 570 nm. To know the effects of CoQ_0_ on cells, viability was evaluated as the percentage of viable cells by comparing with vehicle-treated control whose arbitrary value was taken as 100%.

### 2.4. Determination of NO Levels in Cultured Media

The concentration of NO in the culture medium was estimated by using Griess reagents (Sigma-Aldrich, St. Louis, MO), NO being a major stable product which was based on the accumulation of nitrite. 4 × 10^5^ RAW264.7 cells were grown in a 12-well plate in DMEM which contained 5 mM arginine. The cultured cells were pretreated with 2.5-12.5 *μ*M of CoQ_0_ for 1 h and later stimulated for 18 h by LPS (1 *μ*g/mL) or 17 h by LPS (1 *μ*g/mL) followed by ATP (5 mM) for 1 h (total 18 h). Posttreatment, 100 *μ*L supernatant was collected and dissolved in an equal amount of Griess reagents (0.75% sulfanilamide in 0.5 N HCl and 0.075% naphthylethylenediamine dihydrochloride in water = 1 : 1 mixture). Then, by using an ELISA microplate reader, the absorbance was recorded at 540 nm.

### 2.5. Cell Extract Preparation and Western Blot Analysis

1 × 10^6^ RAW264.7 cells/dish were cultured in a 6 cm dish and treated with CoQ_0_ (2.5-10 *μ*M) with or without LPS (1 *μ*g/mL) and ATP (5 mM) for the designated period. Posttreatment, all the cells were detached from the culture dish and washed one time in cold PBS. Then, cytoplasmic, nuclear, and total extracts were prepared following the protocols as given by extraction reagents (Pierce Biotechnology, Rockford, IL, USA). Taking bovine serum albumin as standard, the amount of protein in every sample was calculated using the Bio-Rad protein assay reagent (Bio-Rad, Hercules, CA, USA). Using 8-15% sodium dodecyl sulfate-polyacrylamide gel electrophoresis (SDS-PAGE), an equal volume (50 *μ*g) of denatured protein samples was first electrophoresed and later transferred to polyvinylidene fluoride (PVDF) and left overnight. On a subsequent day, blocking of the membranes was done for 30 min at room temperature by using 5% nonfat dry milk. Following blocking at first, using primary antibodies, the membranes were incubated for 2 h and later with horseradish peroxidase-conjugated goat anti-rabbit or anti-mouse antibody for 2 h (Pierce Biotechnology, Rockford, IL, USA). For measuring band intensities, a densitometric graph was developed by using commercial software (AlphaEase, Genetic Technology Inc., Miami, FL, USA) representing control as 100%.

### 2.6. Caspase-1 Activity Assay

The caspase-1 activity assay was performed at first by scraping RAW264.7 cells in cell lysis buffer and then later adding reaction buffer and YVAD-AFC substrate by following the instructions provided by a commercially available caspase-1 activity assay kit (Abcam, Cambridge, UK).

### 2.7. Immunofluorescence Staining

Prior to culture, 1 × 10^4^ RAW264.7 cells/well were treated with CoQ_0_ (10 *μ*M) for 1 h and then stimulated with LPS (1 *μ*g/mL) for 5 h followed by ATP (5 mM) for 1 h and then seeded in an eight-well glass Tek chamber. Post culture using 2% paraformaldehyde, the cells were fixed for 15 min and then permeabilized with 0.1% Triton X-100 for 10 min and then washed and blocked with 10% FBS in PBS. Primary antibodies of anti-NLRP3 and anti-LC3B were incubated with 1.5% FBS for 2 h and then with fluorescein (FITC) (488 nm)-conjugated secondary antibody for 1 h in 6% bovine serum albumin (BSA). Cells were later stained with 1 *μ*g/mL of DAPI for 5 min and washed with PBS and observed using a confocal microscope (630x magnification) (Leica TCS SP2, Heidelberg, Germany).

### 2.8. Intracellular ROS Production Measurement

Using DCFH_2_-DA, ROS that was generated intracellularly was estimated by a fluorescence spectrophotometer as described previously [47]. In brief, 1 × 10^5^ RAW264.7 cells per well were pretreated in a 24-well plate with CoQ_0_ (10 *μ*M) for 1 h with or without LPS (1 *μ*g/mL) for 5 h followed by ATP (5 mM) for 1 h. Later, 10 *μ*M DCFH_2_-DA was provided to the growth medium and incubated at 37°C for 30 min more. Following incubation, warm PBS washing was done for the cells and using fluorescence microscopy (Olympus, Center Valley, PA, USA); thus, generated ROS was measured by observing the alterations in fluorescence which was caused by DCF production by the oxidation of DCFH_2_ [48]. The ROS generated was estimated in fold increase compared with the vehicle-treated cells, which were arbitrarily considered as 1-fold.

### 2.9. Transient siRNA Transfection for LC3B or Nrf2 Silencing

siRNA for silencing LC3B or Nrf2 was transfected in RAW264.7 cells using Lipofectamine RNAiMAX (Invitrogen, Grand Island, NY, USA). In order to carry out transfection, RAW264.7 cells were cultured using a 6-well plate with DMEM having 10% FBS. Before carrying out transfection, the cells were grown to 60% confluence. The culture medium was then replaced with 500 *μ*L of Opti-MEM on the next day, and the cells were transfected with the RNAiMAX transfection reagent. 5 *μ*L RNAiMAX and 250 *μ*L of Opti-MEM were mixed and incubated together at room temperature for 5 min. In another tube, siRNA (100 pM) was prepared and added to the tube containing 250 *μ*L of Opti-MEM. Thus, the obtained mixture was added to the diluted RNAiMAX. The siRNA/RNAiMAX mixture (500 *μ*L) was allowed to incubate 25 min extra at room temperature to form a transfection complex. Thus, the obtained complex was subsequently added to the 6-well plate—making 1 mL as the final transfection volume. 6 h posttransfection, the medium was substituted by 2 mL standard culture medium and grown at 37°C. Ultimately, the cells were incubated along with CoQ_0_ (10 *μ*M) for 1 h with or without LPS (1 *μ*g/mL) for 5 h followed by ATP (5 mM) for 1 h. Expression of LC3-I/II and pro-IL1*β* was quantified.

### 2.10. Statistical Analysis

All the results were expressed as the mean ± standard deviation (SD). Analysis of variance (ANOVA) followed by Dunnett's pair-wise comparisons was used for the analysis of all data. ^∗^*p* < 0.05,  ^∗∗^*p* < 0.01, and^∗∗∗^*p* < 0.001 when compared with untreated control cells, and ^#^*p* < 0.05, ^##^*p* < 0.01, and ^###^*p* < 0.001 when compared with 3-methyladenine (3-MA), or LPS/ATP-stimulated cells were considered to be statistically significant.

## 3. Results

### 3.1. The Effects of Coenzyme Q_0_ (CoQ_0_) on Cell Viability of RAW264.7 Macrophages

**Figure 1:**
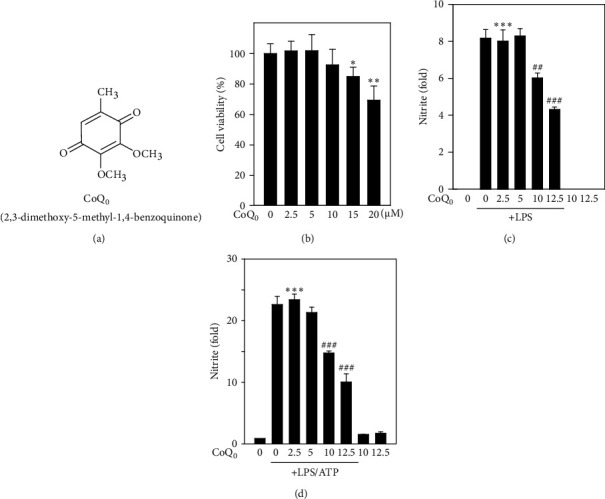
Coenzyme Q_0_ (CoQ_0_) suppresses NO production in LPS- or LPS/ATP-stimulated RAW264.7 macrophages. (a) Structure of Coenzyme Q_0_ (CoQ_0_, 2,3-dimethoxy-5-methyl-1,4-benzoquinone). (b) MTT assay carried out by treating RAW264.7 cells with CoQ_0_ (2.5-20 *μ*M) for 24 h. (c, d) Production of NO was estimated by measuring the nitrite formation and stable end-metabolic of NO in the culture medium. Prior to NO estimation, cells were treated with different doses of CoQ_0_ ranging from 2.5 to 12.5 *μ*M for 1 h, and then, LPS (1 *μ*g/mL) was stimulated for 18 h or LPS (1 *μ*g/mL) for 17 h followed by 5 mM ATP treatment for 1 h. The results were calculated as the mean ± SD of three independent experiments. ^∗∗^*p* < 0.01;  ^∗∗∗^*p* < 0.001, compared with untreated control cells, and ^##^*p* < 0.01; ^###^*p* < 0.001 compared with LPS or LPS/ATP-stimulated cells assigned as statistically significant.

In order to investigate the anti-inflammatory, as well as antioxidant, properties of CoQ_0_, first of all, the cytotoxic effects of different doses of CoQ_0_ (Figure 1(a)) ranging from 2.5 to 20 *μ*M on RAW264.7 macrophages were examined. The results of MTT analysis revealed that when treating with CoQ_0_ for 24 h, there was no obvious effect on the viability of macrophage up to 10 *μ*M concentration, but a significant downregulation was observed with 20 *μ*M treatment (Figure 1(b)). So, depending on this result, the non- or subcytotoxic concentrations of CoQ_0_, i.e., ≤15 *μ*M, were taken for further carrying out *in vitro* studies and examining its response on LPS/ATP-induced inflammation and redox imbalance.

### 3.2. CoQ_0_ Suppresses Production of NO in LPS- or LPS/ATP-Stimulated RAW264.7 Macrophages

To investigate the anti-inflammatory properties of CoQ_0_, macrophages were first treated with CoQ_0_ (2.5-12.5 *μ*M) for 1 h and later stimulated either with LPS (1 *μ*g/mL) for 18 h or LPS (1 *μ*g/mL) for (17 h) and then by ATP (5 mM) for 1 h. The results revealed that LPS stimulation alone significantly increased nitric oxide (NO) production in culture medium, but treatment with CoQ_0_ significantly decreased NO production in a dose-dependent fashion (Figure 1(c)), but this effect was reversed when macrophages were LPS/ATP-stimulated (Figure 1(d)). However, there were no significant changes observed with CoQ_0_ alone-treated cells (Figures 1(c) and 1(d)).

### 3.3. CoQ_0_ Inhibits LPS/ATP-Stimulated IL1*β* Expression through NLRP3 Inflammasome and Procaspase-1 Activation in RAW264.7 Macrophages

**Figure 2:**
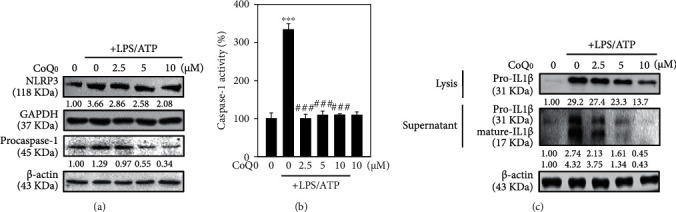
CoQ_0_ inhibits NLRP3 inflammasome activation in LPS/ATP-stimulated RAW264.7 macrophages. Cells were first treated with CoQ_0_ (2.5-10 *μ*M) and later stimulated with LPS (1 *μ*g/mL) for 5 h followed by ATP (5 mM) treatment for 1 h. (a) The expression of NLRP3 and procaspase-1 protein was determined by Western blotting. (b) Caspase-1 activity was measured using a caspase-1 activity assay kit. (c) Pro-IL1*β* expressions in both lysis and supernatant and mature-IL1*β* in the supernatant were determined by Western blot using *β*-actin as internal control as well as band intensities were calculated by AlphaEaseFCTM (Genetic Technologies, Inc., Florida, USA) software. The data were calculated from the mean ± standard deviation (SD) of three independent experiments and ^∗∗∗^*p* < 0.001, compared with untreated control cells, and ^###^*p* < 0.001 compared with LPS/ATP-stimulated cells which was significant.

At first, we analyzed whether CoQ_0_ inhibits activation of NLRP3 inflammasome in RAW264.7 macrophages. For this, we used a well-known *in vitro* model of NLRP3 inflammasome activation, where ATP propels cleavage of caspase-1 in macrophages that are LPS primed [49]. RAW264.7 cells were pretreated with LPS and later stimulated with ATP along with different concentrations of CoQ_0_. Here, we investigated whether CoQ_0_ could repress the LPS/ATP-induced NLRP3 and procaspase-1 activation in macrophages. As anticipated and proven by the Western blot assay, the procaspase-1 was noticeably increased in macrophages stimulated with LPS/ATP corroborating that NLRP3 inflammasome was activated (Figure 2(a)). CoQ_0_ treatment significantly suppressed NLRP3 and procaspase-1 in a dose-dependent manner (Figure 2(a)), and the reduced activity of caspase-1 was observed (Figure 2(b)). To further prove the anti-inflammatory effects of CoQ_0_, using the Western blot assay, we examined the expression of pro-IL1*β* in lysis fractions and mature and pro-IL1*β* in supernatant fractions of macrophages. Upon LPS/ATP stimulation, there was a significant upregulation in the expression of pro-IL1*β* in lysis and both mature and pro-IL1*β* in supernatant fractions which were substantially inhibited by the treatment of CoQ_0_ (2.5-10 *μ*M, 1 h) in a dose-dependent manner (Figure 2(c)).

### 3.4. CoQ_0_ Increases the Accumulation of LC3-II and Activates Autophagy in LPS/ATP-Stimulated RAW264.7 Macrophages

**Figure 3:**
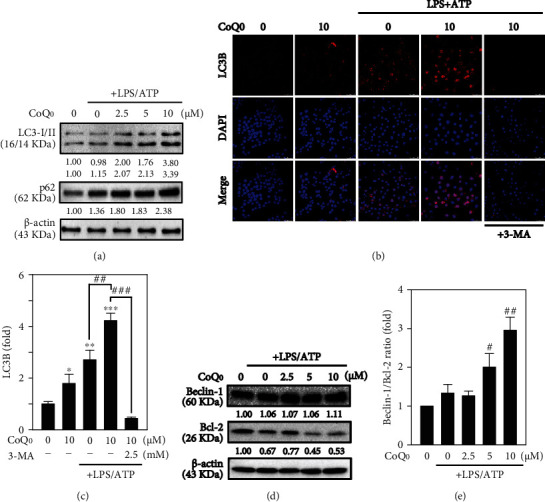
CoQ_0_ induces autophagy in LPS/ATP-stimulated RAW264.7 macrophages. Cells were first treated with 2.5-10 *μ*M of CoQ_0_ and/or 2.5 mM 3-MA (autophagy inhibitor) for 1 h and then followed by stimulation of LPS (1 *μ*g/mL) for 5 h and ATP (5 mM) for 1 h. (a) The expression of LC3-I/LC3-II and p62/SQSTM1 protein was estimated by Western blot. (b) The modifications in LC3B expression were observed by immunofluorescence staining. Cells were incubated with anti-LC3B antibody followed by secondary antibody labelled with FITC. 630x magnification of a confocal microscope was used to visualize the subcellular localization of LC3B. (c) Fold changes in LC3B were determined. (d) CoQ_0_ affects Beclin-1 and Bcl-2 expression in a dose-dependent manner shown by Western blot analysis. (e) Beclin-1/Bcl-2 ratio relative changes were measured by commercial software representing control as 1-fold. The results were calculated as the mean ± SD of three independent experiments. ^∗^*p* < 0.05;  ^∗∗^*p* < 0.01;  ^∗∗∗^*p* < 0.001, compared with untreated control cells, and ^#^*p* < 0.05; ^##^*p* < 0.01; ^###^*p* < 0.001 compared with 3-MA- or LPS/ATP-stimulated cells assigned statistically significant.

LC3-II is a well-known marker for autophagy that reveals the lysosomal turnover and autophagy inside the cells, so monitoring of LC3-II is essential to know about autophagy. Methods such as Western blot and immunofluorescence are widely used for detecting autophagy in cells [50]. Thus, we investigated if the inhibition of NLRP3 inflammasome by CoQ_0_ is regulated by the induction of autophagy. Recently, it has been disclosed that p62/SQSTM1 remains at the interface that links autophagy along with oxidative stress signaling [51]. The expression of p62, which is also known as sequestosome 1 (SQSTM1), is used to measure the autophagy flux [52]. It is responsible for the proteasomal degradation of ubiquitinated proteins and is found all over the cell as well as in various signaling pathways. During autophagy, this protein binds with LC3 through a specific motif and degrades itself [53]. CoQ_0_ (2.5-10 *μ*g/mL) along with LPS/ATP stimulation in RAW264.7 macrophages and its effect on the expression of LC3-I/II and p62 were explored in this study. Western blot data revealed that with increasing concentrations of CoQ_0_, LC3-I/II and p62 expression went on increasing (Figure 3(a)). Employing the fluorescence method, accumulation of LC3 in RAW264.7 cells was further investigated. CoQ_0_ (10 *μ*g/mL) upregulated LC3 without or with LPS/ATP-stimulated RAW264.7 cells which was consistent with Western blot results (Figure 3(b)). This effect was found to be statistically significant and measured approximately 3- or 5-fold without or with LPS/ATP stimulation-treated cells in comparison to control cells (Figure 3(c)). Additionally, to examine the role of CoQ_0_ (10 *μ*g/mL) mediating autophagy in LPS/ATP-stimulated RAW264.7 cells, 3-MA was used which being a pharmacological inhibitor of autophagy interrupts the lysosomal function throughout autophagy. The fluorescence data revealed that CoQ_0_ alone treatment exhibited that 3-MA (2.5 mM) inhibited the LC3-II accumulation in the early autophagy (Figures 3(b) and 3(c)). These results suggested that CoQ_0_ activated autophagy through LC3-II signaling cascades in LPS/ATP-stimulated RAW264.7 macrophages.

### 3.5. CoQ_0_ Dysregulates Beclin-1 and Bcl-2 Ratio Leading to Autophagy in LPS/ATP-Stimulated RAW264.7 Macrophages

Beclin-1 is one of the important proteins to initiate autophagy which recruits crucial autophagy proteins to a preautophagosomal structure. In addition, Bcl-2 combines with Beclin-1 and decreases its proautophagy profile, but the apoptotic role cannot be neutralized [54]. The effects of various doses of CoQ_0_ in Beclin-1 and Bcl-2 protein expression were examined through Western blot analysis which demonstrated that CoQ_0_ (2.5-10 *μ*M for 6 h) dose-dependently suppressed Bcl-2 expression in LPS/ATP-stimulated RAW264.7 macrophages (Figures 3(d) and 3(e)). In contrast, Beclin-1 expression did not show significant changes. Remarkably, due to low Bcl-2 expression, CoQ_0_ dysregulated Beclin-1 and Bcl-2 protein ratio thus guiding to autophagy (Figures 3(d) and 3(e)).

### 3.6. CoQ_0_ Reduces the Phosphorylation of PI3K/AKT, p70 S6 Kinase, and mTOR Expressions Leading to Autophagy in LPS/ATP-Stimulated RAW264.7 Macrophages

**Figure 4:**
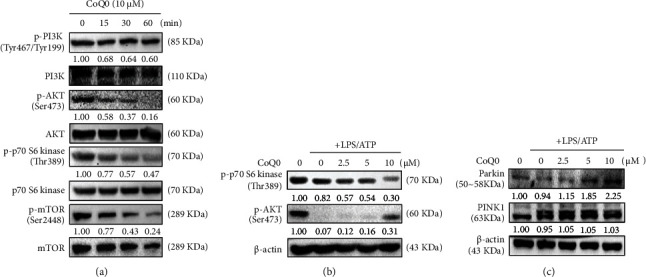
CoQ_0_ induces mitophagy in LPS/ATP-stimulated RAW264.7 macrophages. (a) Time-dependent expression of p-PI3K, PI3K, p-AKT, AKT, p-p70 S6 kinase, p70 S6 kinase, p-mTOR, and mTOR was determined by Western blot. Cells were first treated with CoQ_0_ (10 *μ*M) for 0-60 min and then stimulated with LPS (1 *μ*g/mL) for 5 h followed by ATP (5 mM) for 1 h. (b) Dose-dependent expression of p-p70 S6 kinase and p-AKT was determined by Western blot. Cells were pretreated with CoQ_0_ (2.5-10 *μ*M) for 60 min and then LPS (1 *μ*g/mL) stimulation for 5 h followed by ATP (5 mM) for 1 h, and the changes in the intensities of protein bands were measured by commercial quantitative software. (c) The cells were first treated with CoQ_0_ (2.5-10 *μ*M) for 60 min and then stimulated with LPS (1 *μ*g/mL) for 5 h followed by ATP (5 mM) for 1 h, and lastly, expression of Parkin and PINK1 was determined by Western blot.

The PI3K/AKT/p70 S6 kinase/mTOR signaling pathway is a key regulator and affects the regulation of autophagy [55]. In this study, the effects of CoQ_0_ on PI3K/AKT/P70 S6 kinase/mTOR expression in LPS/ATP-stimulated RAW264.7 macrophages were investigated. In comparison to untreated control, for CoQ_0_ (10 *μ*g/mL) treated for 0-60 min, the expression of phosphorylated PI3K (Tyr467/Tyr199), AKT (Ser473), p70 S6 kinase (Thr389), and mTOR (Ser2448) was downregulated in a time-dependent manner (Figure 4(a)). Additionally, for CoQ_0_ (2.5-10 *μ*g/mL) treated for 60 min, the expression of p70 S6 kinase (Thr389) and AKT (Ser473) was downregulated in a dose-dependent manner (Figure 4(b)). So, the results revealed that the inhibition of PI3K/AKT/p70 S6 kinase/mTOR was due to autophagy activation by CoQ_0_ in LPS/ATP-induced RAW264.7 macrophages.

### 3.7. CoQ_0_ Induces Mitophagy in LPS/ATP-Stimulated RAW264.7 Macrophages

Cells remove defective mitochondria through a special type of autophagy known as mitophagy [56]. Evidence showed that Parkin is recruited from the cytosol to damaged mitochondria to regulate the removal of the damaged organelles [21]. In order to know whether Parkin was recruited into the mitochondria, Western blot analysis was performed. CoQ_0_ treatment reduced the Parkin expression hence suggesting that it impaired the mitochondria (Figure 4(c)). But, in cells pretreated with CoQ_0_, there were no significant changes observed in PINK1 expression (Figure 4(c)), thus suggesting that CoQ_0_ pretreatment upregulated the expression of Parkin protein to recruit the damaged mitochondria.

### 3.8. CoQ_0_ Inhibits LPS/ATP-Stimulated NLRP3 Inflammasome Activation through Autophagy Induction in RAW264.7 Macrophages

**Figure 5:**
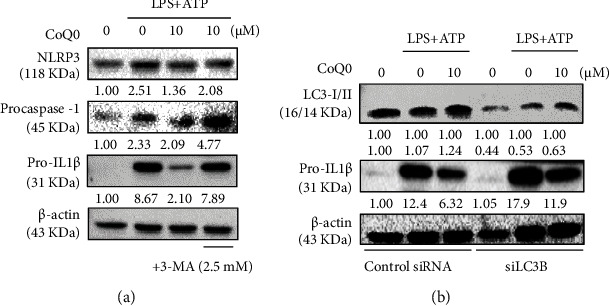
CoQ_0_ inhibits NLRP3 inflammasome activation through autophagy induction in LPS/ATP-stimulated RAW264.7 macrophages. (a) Cells were pretreated with CoQ_0_ (10 *μ*M) and/or autophagy inhibitor 3-MA (2.5 mM) for 1 h and then stimulated with LPS (1 *μ*g/mL) for 5 h followed by ATP (5 mM) for 1 h. NLRP3, procaspase-1, pro-IL1*β*, and LC3-I/II were determined by Western blot. (b) LC3 knockdown attenuated the protective effects of CoQ_0_. Cells were first transfected with siRNA that is specific to either LC3B or a nonsilencing control then pretreated with CoQ_0_ (10 *μ*M) for 1 h and then stimulated with LPS (1 *μ*g/mL) for 5 h followed by ATP (5 mM) for 1 h, and the expression of LC3-I/II or pro-IL1*β* proteins in both control and siLC3B was determined using Western blot analysis.

In order to know if there was a role of autophagy in NLRP3 inflammasome suppression, an autophagy inhibitor, 3-MA, which blocks the formation of autophagosome was supplied to the culture medium. Remarkably, 3-MA reversed the effects of CoQ_0_ in reducing the expressions of NLRP3, procaspase-1, and pro-IL1*β* which was significantly upregulated in LPS/ATP-stimulated macrophages due to activation of NLRP3 inflammasome (Figure 5(a)). Furthermore, siLC3B reversed the results of CoQ_0_ in failing to suppress pro-IL1*β* expression when autophagy was suppressed by LC3 silencing or knockdown (Figure 5(b)). Hence, the data indicated that autophagy acts as a cell-intrinsic phenomenon to restrict the activation of NLRP3 inflammasome, and by inducing autophagy, CoQ_0_ potentiates this regulatory mechanism.

### 3.9. CoQ_0_ Attenuates LPS/ATP-Stimulated ROS Generation in RAW264.7 Macrophages

**Figure 6:**
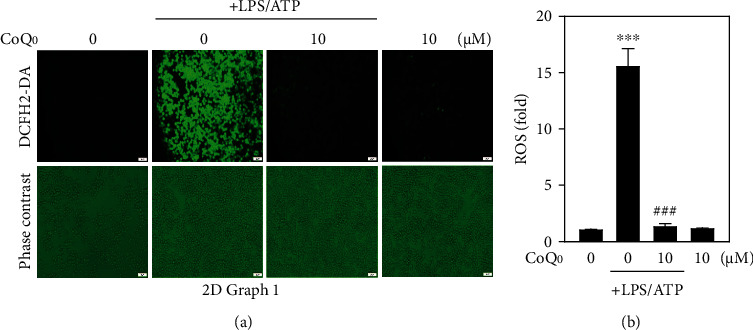
CoQ_0_ attenuates LPS/ATP-stimulated ROS generation in RAW264.7 macrophages. (a) Cells were pretreated with CoQ_0_ (10 *μ*M) for 1 h and then stimulated with LPS (1 *μ*g/mL) for 5 h followed by ATP (5 mM) for 1 h. The level of intracellular ROS was measured by DCF fluorescence using fluorescence microscopy (200x magnification). (b) Data are presented as fold change, and the results were calculated as the mean ± SD of three experiments, where ^∗∗∗^*p* < 0.001, compared with untreated control cells, and ^###^*p* < 0.001 compared with LPS/ATP-stimulated cells.

ROS plays a crucial role in the regulation of different inflammatory mediators. The accumulation of LPS-stimulated ROS in macrophages can increase the inflammatory responses [57]. When LPS/ATP was stimulated for 6 h to the RAW264.7 macrophages, it triggered the intracellular ROS generation as shown in Figures 6(a) and 6(b). But treatment with CoQ_0_ (10 *μ*M) for 1 h earlier to LPS/ATP stimulation significantly reduced the ROS production despite the cells incubated with CoQ_0_ alone, i.e., without stimulation, revealing that there were no changes in ROS levels when compared to control (Figures 6(a) and 6(b)).

### 3.10. CoQ_0_ Inhibits ROS-Mediated NLRP3 Inflammasome Activation and IL1*β* Expression in LPS/ATP-Stimulated RAW264.7 Macrophages

**Figure 7:**
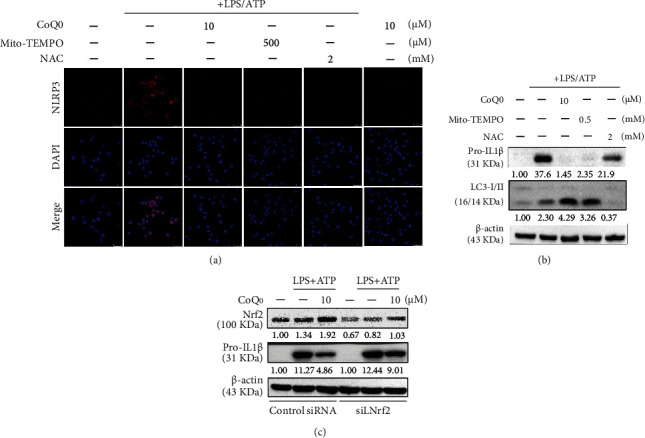
CoQ_0_ inhibits ROS-mediated NLRP3 inflammasome activation through autophagy induction and Nrf2 activation in LPS/ATP-stimulated RAW264.7 macrophages. Cells were pretreated with CoQ_0_ (2.5-10 *μ*M), Mito-TEMPO (0.5 mM), or NAC (2 mM) for 1 h and then stimulated with LPS (1 *μ*g/mL) for 5 h followed by ATP (5 mM) for 1 h. (a) Immunofluorescence staining of RAW264.7 cells and the nuclear localization of NLRP3 were visualized by the immunofluorescence method. Cells were stained with DAPI (1 *μ*g/mL) for 5 min and examined by fluorescence microscopy. (b) The expression of pro-IL1*β* or LC3-I/II proteins was measured by Western blot analysis. (c) Nrf2 knockdown attenuated the protective effects of CoQ_0_. Cells were transfected with siRNA that is specific to either Nrf2 or a nonsilencing control. Transfected cells were pretreated with CoQ_0_ (2.5-10 *μ*M) for 1 h and then stimulated with LPS (1 *μ*g/mL) for 5 h followed by ATP (5 mM) for 1 h, and the expression of Nrf2 or pro-IL1*β* proteins in both control and siNrf2 was measured by Western blot analysis.

Recent evidence suggests that mitochondria-derived reactive oxygen species (mtROS) are linked to IL1*β* expression through the Nod-like receptor pyrin domain-containing 3 (NLRP3) inflammasome, which is a redox sensor [58]. NLRP3 inflammasome activation in response to LPS and ATP needs mtROS produced from defective mitochondria, and mitochondrial DNA is released into the cytosol in an NLRP3- and mtROS-dependent manner [59]. The expression of NLRP3 was monitored using a fluorescence microscope. We found that CoQ_0_ (10 *μ*M), Mito-TEMPO (0.5 mM), and NAC (2 mM) treatment suppressed NLRP3 expression in LPS/ATP-induced RAW264.7 macrophages (Figure 7(a)). Likewise, the expression of pro-IL1*β* was estimated using Western blot. CoQ_0_, Mito-TEMPO, and NAC treatment inhibited the expression of pro-IL1*β* in comparison to LPS/ATP alone treatment. The effects of CoQ_0_ and Mito-TEMPO were found to be significant in comparison to NAC treatment (Figure 7(b)). These results indicated that ROS including mtROS signaling cascades are involved in the regulation of CoQ_0_-inhibited NLRP3 inflammasome and pro-IL1*β* expression.

### 3.11. CoQ_0_ Inhibits mtROS-Mediated NLRP3 Inflammasome Activation through Mitophagy Induction in LPS/ATP-Stimulated RAW264.7 Macrophages

Mitophagy is a mitochondria-selective autophagic mechanism that exists within cells to remove damaged mitochondria and preserve mitochondrial homeostasis in the face of stress [60]. During mitophagy, mitochondria are engulfed into the vesicles which are coated with autophagosomal marker MAP1 light chain 3 (LC3) [61]. Blocking mitophagy triggers an accumulation of damaged, ROS-producing mitochondria, which activates the NLRP3 inflammasome [49]. In order to confirm the influence of mitophagy, cells were first treated with CoQ_0_, Mito-TEMPO, or NAC, and then, changes in expression of LC3-II were determined by Western blot. Our analysis depicted that LPS/ATP-induced LC3-II expression was significantly enhanced by CoQ_0_ and Mito-TEMPO, but this effect was not observed in NAC treatment (Figure 7(b)). These findings exhibited that mitophagy is involved in the regulation of CoQ_0_-inhibited NLRP3 inflammasome activation and pro-IL1*β* expression in LPS/ATP-stimulated RAW264.7 macrophages.

### 3.12. Nrf2 Knockdown Suppresses CoQ_0_ Mediated Anti-NLRP3 Inflammasome Activation

In our previous study, CoQ_0_ increased Nrf2 nuclear translocation and Nrf2/ARE-signaling in LPS-stimulated macrophages [29]. Recent studies have revealed that Nrf2 could negatively regulate the activity of NLRP3 inflammasome by suppressing activation of NLRP3 inflammasome induced by ROS [62]. Recently, it has been demonstrated that pro-IL1*β* is required for autophagic degradation [63]. We addressed this possibility, by quantifying pro-IL1*β* in LPS/ATP-stimulated lysates of macrophages treated with CoQ_0_ by Western blot. Cells were first transfected with Nrf2-specific siRNA or a nonsilencing control and posttransfection treated with 10 *μ*M of CoQ_0_ for 1 h and then stimulated with LPS (1 *μ*g/mL) for 5 h and ATP (5 mM) for 1 h, and the expression of Nrf2 or pro-IL1*β* proteins in control as well as siNrf2 was estimated via Western blot. When Nrf2 was knocked down using siRNA, it attenuated the protective effects of CoQ_0_. CoQ_0_ (10 *μ*M) treatment decreased the level of pro-IL1*β* in macrophages when Nrf2 is silenced in comparison to control siRNA (Figure 7(c)) suggesting that CoQ_0_-mediated anti-NLRP3 inflammasome activation was suppressed because of Nrf2 knockdown.

## 4. Discussions

Earlier, we have shown that CoQ_0_ (2,3-dimethoxy-5-methyl-1,4-benzoquinone) regulated NF*κ*B/AP-1 activation and enhanced Nrf2 stabilization in the attenuation of LPS-induced inflammation and redox imbalance [29]. The noncytotoxic concentrations of CoQ_0_ (2.5-10 *μ*M) inhibited iNOS/COX-2 protein expression in LPS-stimulated macrophages, resulting in lower NO, PGE2, INF, and IL1 secretions. Moreover, in LPS-stimulated macrophages, CoQ_0_ induced HO-1 and NQO-1 gene expression by increasing Nrf2 nuclear translocation and Nrf2/ARE signaling [29]. To our knowledge, this is the first study that CoQ_0_ inhibited ROS-mediated NLRP3 inflammasome activation through mitophagy induction and Nrf2 activation in LPS/ATP-stimulated macrophages.

LPS is a potent activator of monocytes and macrophages that triggers the secretion of several proinflammatory cytokines [37]. We first tested if CoQ_0_ could exhibit an anti-inflammatory effect on LPS/ATP-stimulated macrophages. Data suggested that CoQ_0_ dose-dependently and significantly decreased NO production and inferred the protective effect of CoQ_0_ on macrophages. The inflammasome NLRP3 is a multiprotein complex that regulates caspase-1 activation and assists in releasing proinflammatory cytokine IL1*β* [64, 65], one of the most characterized cytokines known to play an important role in autoimmune diseases [64, 65]. Our data revealed that LPS/ATP alone-induced macrophage showed the increased expression of NLRP3 inflammasome and procaspase-1; however, CoQ_0_ treatment could repress the LPS/ATP-induced NLRP3 and procaspase-1 activation. Caspase-1 also called as an interleukin-1*β* converting enzyme (ICE) is a proteolytic enzyme that processes the mature form of the inactive precursor IL1*β* [66, 67]. The inflammasome assembly of NLRP3 and subsequent self-processing of proteolytics will activate caspase-1 itself as an inactive cytoplasm precursor [68]. The caspase-1 activity measurement assay revealed that LPS/ATP stimulation on macrophages increased caspase activation, which was then decreased by CoQ_0_ treatment. Likewise, increased expression of pro-IL1*β* and mature IL1*β* in lysis and supernatant and pro-IL1*β* in lysis was observed in LPS/ATP-stimulated macrophages. However, this effect was found to be downregulated with increasing dose treatment of CoQ_0_ thus suggesting that CoQ_0_ can inhibit LPS/ATP-stimulated NLRP3 inflammasome activation in RAW264.7 macrophages. Our data revealed that CoQ_0_ regulates the activation of macrophages by enhancing a negative regulatory loop amongst NLRP3 inflammasome.

As LC3 is a promising autophagy marker [69], its intracellular distribution has been examined to find out whether CoQ_0_ can induce autophagy in LPS/ATP-stimulated RAW264.7 macrophages. Our results revealed that the accumulation of LC3-I and LC3-II was dose-dependently increased following CoQ_0_ treatment. The high dose (10 *μ*M) of CoQ_0_ notably increased with the high accumulation of LC3-II which suggested that CoQ_0_ activated autophagy through LC3-II signaling cascades in LPS/ATP-stimulated RAW264.7 macrophages. Recently, p62/SQSTM1 is found to be at the interface that links autophagy as well as oxidative stress [51]. p62 or sequestosome 1 (SQSTM1) is an important protein that binds directly to LC3 and then undergoes self-degradation while autophagy occurs [70]. Being a multifunctional ubiquitin-binding protein, p62/SQSTM1 involves many important processes of autophagy [71]. p62/SQSTM1 plays a crucial part in the oxidative stress response pathway through its direct association with the ubiquitin ligase adaptor Kelch-like ECH-associated protein 1 (Keap-1), resulting in Nrf2 activation [51]. p62/SQSTM1 being an autophagy adaptor combines with protein aggregates which are ubiquitinated and convey them into the autophagosome thus enhancing selective autophagy [72]. Furthermore, p62/SQSTM1 in recent time appeared as a mediator of the Nrf2-Keap-1-ARE (antioxidant response element) axis by competing with the relationship between Nrf2 and Keap-1 as well as activating Nrf2, the target genes of which are antioxidant proteins and detoxification enzymes [73]. From our results, it was known that the expression level of p62/SQSTM1 remarkably increased with CoQ_0_ (2.5-10 *μ*M) incubation after 24 h in a dose-dependent fashion suggesting that CoQ_0_ induced autophagy in LPS/ATP-stimulated RAW264.7 macrophages. The increase in p62/SQSTM1 levels was linked with the increasing accumulation of LC3 in RAW264.7 macrophages, and p62/SQSTM1 had an important role in regulating the CoQ_0_ action in suppression of NLRP3 inflammasome.

The proteins of the Bcl-2 family serve as key regulators of mitochondrial-mediated apoptosis and work as either activators or inhibitors [74]. The relationship between Beclin-1 and Bcl-2 is complex, and the Beclin-1 proautophagic property can be reduced by Bcl-2 [75]. Hence, the effect of CoQ_0_ on Bcl-2 protein and its function in Beclin-1 expression in LPS/ATP-stimulated RAW264.7 macrophages was studied. Western blotting data revealed that Beclin-1 proteins dramatically increased with CoQ_0_ in a dose-dependent manner. In contrast, Bcl-2 expression was suppressed with CoQ_0_. The increase in the ratio of Beclin-1/Bcl-2 with increasing concentration of CoQ_0_ suggested that there was an autophagy induction in LPS/ATP-stimulated macrophages. Pretreatment of cells with 3-MA (2.5 mM) successfully decreased LC3B indicating that autophagy was caused by CoQ_0_.

Later, we demonstrated the signaling pathways leading to the CoQ_0_-mediated effects on LPS/ATP-stimulated RAW264.7 macrophages. The PI3K/AKT, p70 S6 kinase, and mTOR pathways are critical for inflammation and different diseases [76]. mTOR collaborates with PI3K effectors to phosphorylate the p70 S6 kinase which is associated with protein translation of an mRNA transcript family that encodes the fundamental components of protein synthesis apparatus [77]. Our data indicated that CoQ_0_ (10 *μ*M) time- and dose-dependently downregulated the phosphorylations of PI3K (Tyr467/Tyr199)/AKT (Ser473), p70 S6 kinase (Thr389), and/or mTOR (Ser 2448) proteins in LPS/ATP-stimulated cells inferring that autophagy plays a critical role in CoQ_0_-mediated effects on RAW264.7 macrophages (Figure 4).

Using a specialized type of autophagy also called mitophagy, cells eliminate defective mitochondria [56]. Evidence has demonstrated that Parkin is recruited from the cytosol to depolarized mitochondria to guide the elimination of the damaged organelles also known as selective autophagy or mitophagy [21]. The PINK1 and E3 ubiquitin ligase Parkin pathway mediate the removal of damaged mitochondria. Accumulation of Parkin and PINK1 takes place in impaired mitochondria to boost their segregation from the mitochondrial network and target these organelles for autophagic degradation in a process that involves ubiquitination of Parkin-dependent mitochondrial proteins [56]. Our analysis showed that Parkin expression was reduced by the treatment of CoQ_0_ indicating that it impaired the mitochondria. However, there were no significant changes observed in PINK1 expression when pretreated with CoQ_0_ thus suggesting that CoQ_0_ pretreatment upregulated the expression of Parkin protein to recruit the damaged mitochondria. We exhibited that CoQ_0_ has mitigated LPS/ATP-induced inflammation along with redox imbalance in RAW264.7 macrophages. This result was similar to our previous study which showed that induction of LPS in RAW264.7 cells increased levels of ROS [57]. To know whether there was any function of autophagy in NLRP3 inflammasome suppression, we treated the LPS/ATP-stimulated RAW264.7 macrophages with autophagy inhibitor (3-MA) which blocks autophagosome formation. Notably, 3-MA reversed the effects of CoQ_0_ on reducing the NLRP3 inflammasome, procaspase-1, and pro-IL1*β* expression. Additionally, when autophagy was suppressed by LC3 silencing, siLC3 reversed the results of CoQ_0_ in failing to suppress pro-IL1*β* expression. So, from this result, we came to know that mitophagy acts as a cell-intrinsic phenomenon to restrict NLRP3 inflammasome activation and CoQ_0_ potentiates this regulatory mechanism through mitophagy induction.

Immune cells such as monocytes and macrophages respond first to any injury in tissue by recognizing danger signals and triggering the inflammatory process [78]. Evidence showed that cell-autonomous regulatory feedback loops are employed in NLRP3 inflammasome regulation, of which autophagy is the most dominant. Former studies have manifested that NLRP3 inflammasome activation is limited by the induction of autophagy of inflammatory signals because of the removal of damaged mitochondria and prevention of mitochondrial ROS release [49]. Consistent with this existing evidence, our study revealed that a noncytotoxic concentration of CoQ_0_ is capable of suppressing intracellular ROS generation and NLRP3 inflammasome against LPS/ATP stimulation in RAW264.7 macrophages. Stress and inflammatory conditions are associated with both the Nrf2 transcription factor and NLRP3 inflammasome, while inflammatory activation of NLRP3 induces inflammation and eventually death of inflammation-activating cells. Nrf2 activation promotes cell survival and prevents inflammation [17]. Nrf2, a transcription factor, activators result in cytoprotective proteins and enzyme expression that, under stress conditions, facilitates the survival of Nrf2-activating cells [17]. In response to LPS and ATP, NLRP3 inflammasome activation requires mitochondrial ROS generated from dysfunctional mitochondria, and mitochondrial DNA is secreted into the cytosol in both NLRP3 and mitochondrial ROS-dependent ways [59]. Mito-TEMPO, which is a mitochondria-specific ROS scavenger, suppressed both IL1*β* secretion after nigericin or ATP exposure [79]. In our study, when LPS/ATP-stimulated RAW264.7 macrophages were treated with CoQ_0_, Mito-TEMPO, and NAC, a significant reduction of LPS/ATP-stimulated NLRP3 inflammasome activation and pro-IL1*β* expression was observed which indicates that ROS signaling cascades are involved in CoQ_0_-inhibited NLRP3 inflammasome activation and pro-IL1*β* expression. Surprisingly, when LPS/ATP-stimulated RAW264.7 macrophages were treated with CoQ_0_ or Mito-TEMPO, but not NAC, there is significantly increased LPS/ATP-induced LC3-II accumulation indicating the occurrence of mitophagy. Following LPS/ATP stimulation, Nrf2 knockdown significantly decreased pro-IL1*β* expression in RAW264.7 macrophages indicating that CoQ_0_ inhibited ROS-mediated NLRP3 inflammasome activation and pro-IL1*β* expression was suppressed due to the Nrf2 activation (Figure 7). From the above, we proposed that CoQ_0_ negatively regulates macrophage activation through mitophagy induction and Nrf2 pathways that hinder a positive feedback loop of NLRP3 inflammasome mechanisms.

## 5. Conclusions

**Figure 8:**
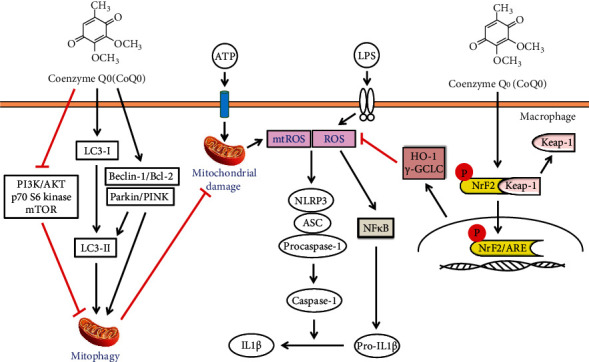
Graphical summary. Collectively, our results showed that subcytotoxic treatments of macrophages with CoQ_0_ displayed antioxidant and anti-inflammatory properties. CoQ_0_ inhibited the NLRP3 inflammasome and procaspase-1 activation which in turn suppressed pro-IL1*β* expression levels. On the other hand, CoQ_0_ exposure in LPS/ATP-stimulated macrophages incited autophagy which was evident by the accumulation of LC3-II and p62/SQSTM1 as well as dysregulation of Beclin1/Bcl-2. This was accompanied by reduced phosphorylation of PI3K/AKT, p70 S6 kinase, and mTOR. Besides, CoQ_0_ inhibited ROS-mediated NLRP3 inflammasome activation through mitophagy induction and Nrf2 activation in LPS/ATP-stimulated macrophages. Altogether, we propose that CoQ_0_ might be a promising candidate for the therapeutics of inflammatory abnormalities due to its effective anti-inflammatory as well as antioxidant properties.

In conclusion, our findings demonstrated that non- or subcytotoxic doses of CoQ_0_ exhibited anti-inflammatory and antioxidant properties (Figure 8). CoQ_0_ suppressed the NLRP3 inflammasome and procaspase-1 activation which in turn downregulated pro-IL1*β* expression levels. Accumulation of LC3-II, p62/SQSTM1, and AVO as well as dysregulation of Beclin1/Bcl-2 showed that CoQ_0_ treatment in LPS/ATP-stimulated macrophages induced autophagy. Additionally, CoQ_0_ reduced the expression of phosphorylated PI3K/AKT, p70 S6 kinase, and mTOR thus leading to autophagy. Besides, CoQ_0_ increased Parkin protein to recruit damaged mitochondria and induced mitophagy in LPS/ATP-stimulated RAW264.7 macrophages. Similarly, CoQ_0_ inhibited ROS-mediated NLRP3 inflammasome activation through mitophagy induction and Nrf2 activation in LPS/ATP-stimulated macrophages. Hence, this study showed that CoQ_0_ might be a promising candidate for the therapeutics of inflammatory disorders due to its effective anti-inflammatory as well as antioxidant properties.

## Data Availability

All the data used to support the findings of this study are included within the article and the supplementary information file(s), and they are available from the corresponding author upon request.

## Supplementary Materials

Supplementary Materials The file contains original raw images of Western blot figures in the manuscript.
