# Supplementary material for: Coenzyme Q0 Inhibits NLRP3 Inflammasome Activation through Mitophagy Induction in LPS/ATP-Stimulated Macrophages
Source: Oxid Med Cell Longev. 2022 Jan 7;2022:4266214. doi: 10.1155/2022/4266214 (PMC8759827; doi:10.1155/2022/4266214)

***Supplementary materials*:** Fig S2A Cells were first treated with CoQ_0_ (2.5-10 μM) and later stimulated with LPS (1 μg/mL) for 5 h followed by ATP (5 mM) treatment for 1 h and then the expression of NLRP3 and procaspase-1 protein were determined by Western blotting. Fig S2C Pro-IL1β expressions in both lysis and supernatant and mature-IL1β in the supernatant were determined by Western blot. Fig S3A Cells were first treated with 2.5-10 μM of CoQ_0_, and/or 2.5 mM 3-MA for 1 h and then followed by stimulation of LPS (1 μg/mL) for 5 h and ATP (5 mM) for 1 h and then the expression of LC3-I/LC3-II and p62/SQSTM1 protein was estimated by Western blot. Fig S3D Effect of CoQ_0_ in Beclin-1 and Bcl-2 expression in the dose-dependent manner shown by Western blot analysis. Fig S4A Time-dependent expression of p-PI3K, PI3K, p-AKT, AKT, p-p70 S6 kinase, p70 S6 kinase, p-mTOR, and mTOR was determined by Western blot. Fig S4B Dose-dependent expression of p-p70 S6 kinase and p-AKT was determined by Western blot. Fig S4C the cells were first treated with CoQ_0_ (2.5-10 μM) for 60 min and then stimulated with LPS (1 μg/mL) for 5 h followed by ATP (5 mM) for 1 h and lastly, expression of Parkin and PINK1 was determined by Western blot. Fig S5A cells were pretreated with CoQ_0_ (10 μM) and/or autophagy inhibitor 3-MA (2.5 mM) for 1 h, and then stimulated with LPS (1 μg/mL) for 5 h following by ATP (5 mM) for 1 h and then NLRP3, procaspase-1, and pro-IL1β were determined by Western blot. Fig S5B LC3B knockdown attenuated the protective effects of CoQ_0_. Cells were first transfected with siRNA that is specific to either LC3B or a non-silencing control then pre-treated with CoQ_0_ (10 μM) for 1 h, and then stimulated with LPS (1 μg/mL) for 5 h following by ATP (5 mM) for 1 h and the expressions of LC3-I/II or pro-IL1β proteins in both control and siLC3B were determined using Western blot analysis. Fig S7B cells were pre-treated with CoQ_0_ (2.5-10 μM), Mito-TEMPO (0.5 mM), or NAC (2 mM) for 1 h, and then stimulated with LPS (1 μg/mL) for 5 h following by ATP (5 mM) for 1 h. The expression of pro-IL1β or LC3-I/II protein was measured by Western blot analysis. Fig S7C Nrf2 knockdown attenuated the protective effects of CoQ_0_. Cells were transfected with siRNA that is specific to either Nrf2 or a non-silencing control. Transfected cells were pre-treated with CoQ_0_ (2.5-10 μM) for 1 h, and then stimulated with LPS (1 μg/mL) for 5 h following by ATP (5 mM) for 1 h and the expression of Nrf2 or pro-IL1β proteins in both control and siNrf2 were measured by Western blot analysis.

**
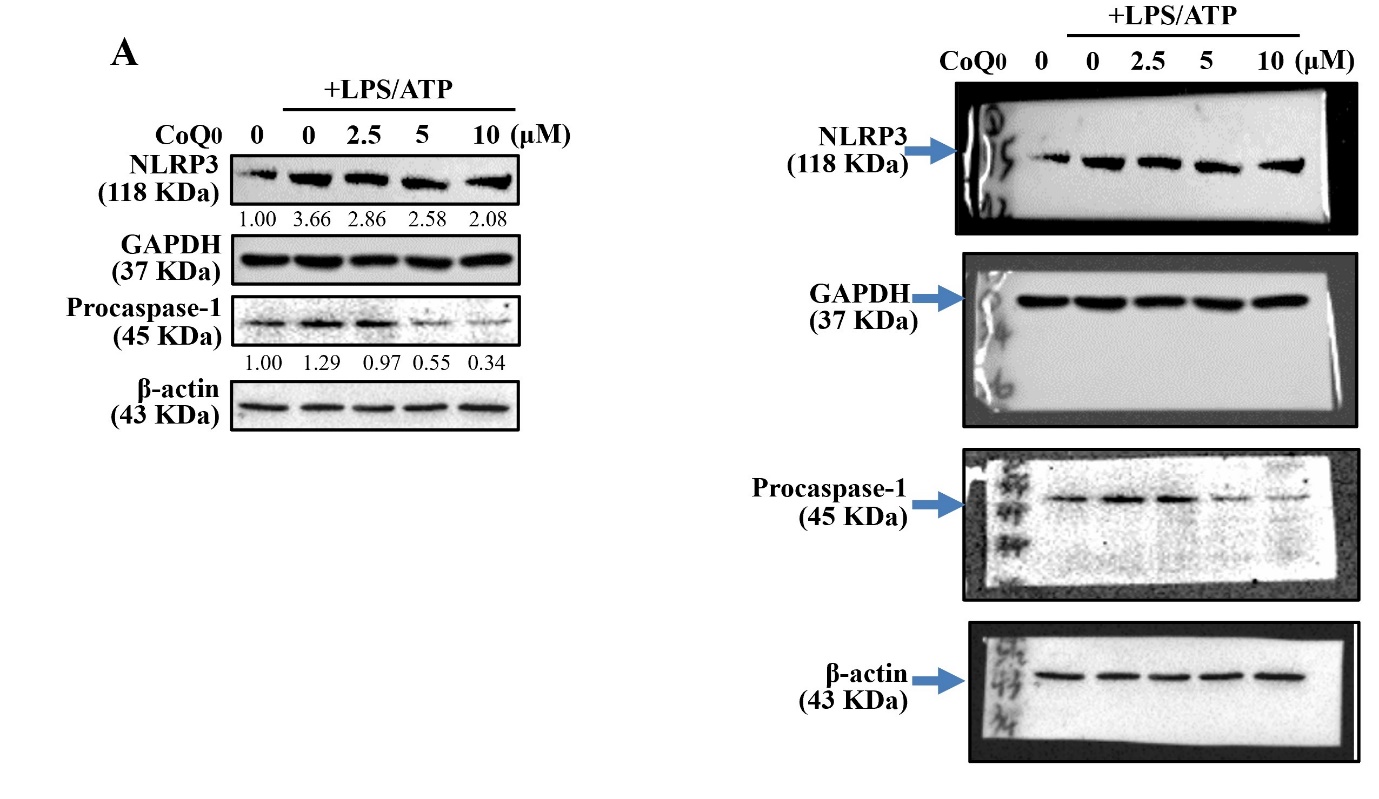
Figure: S2A**

**Figure: S2C**


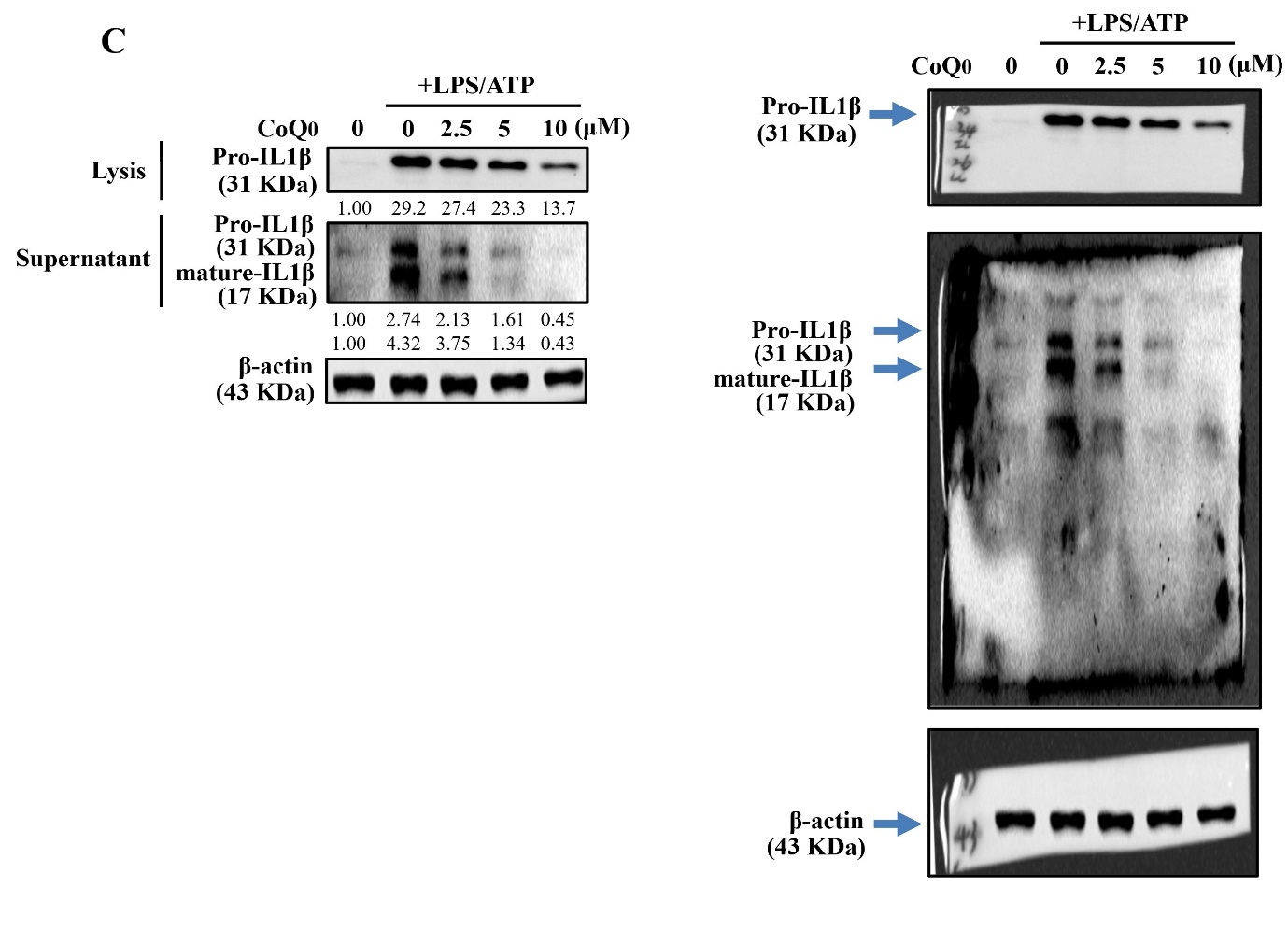


**Figure: S3A**


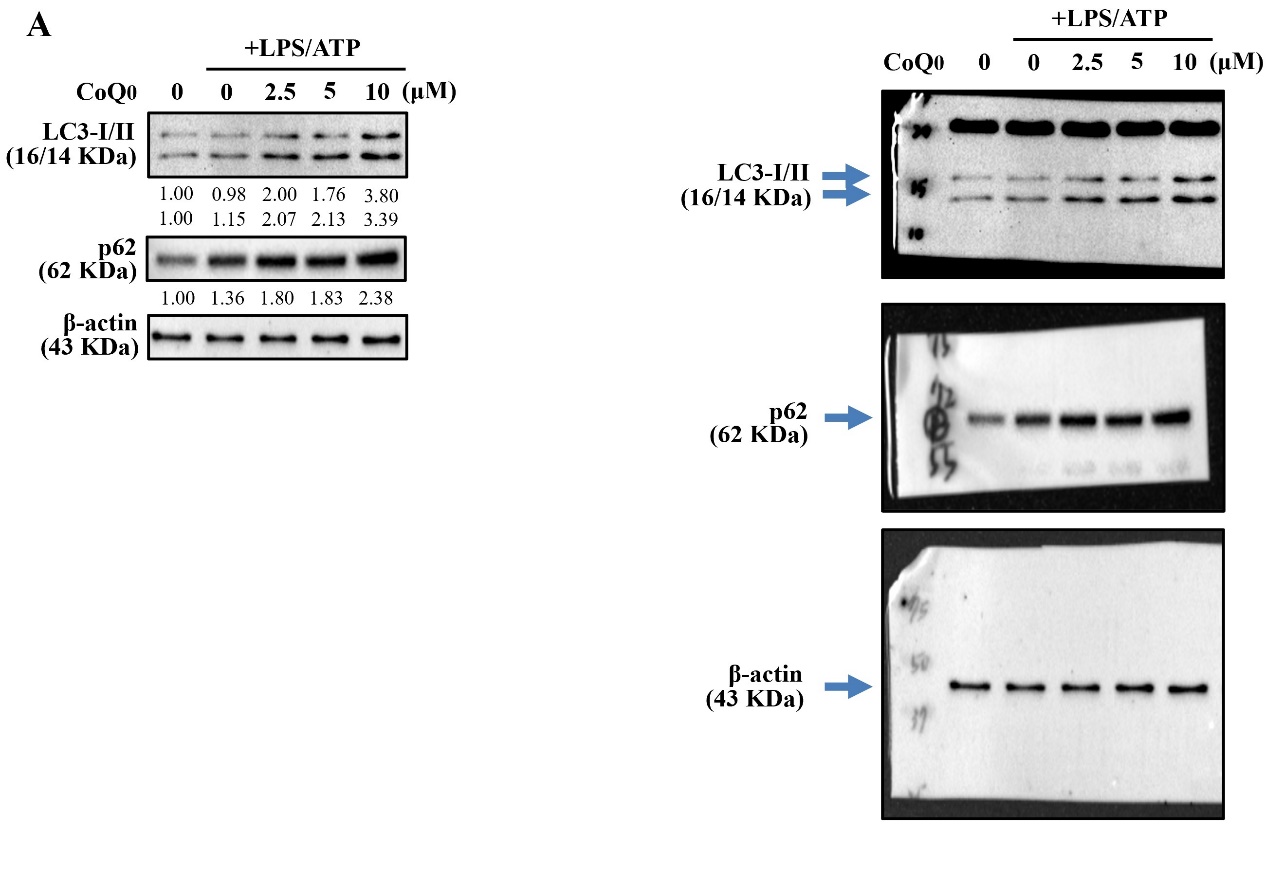


**Figure: S3D**


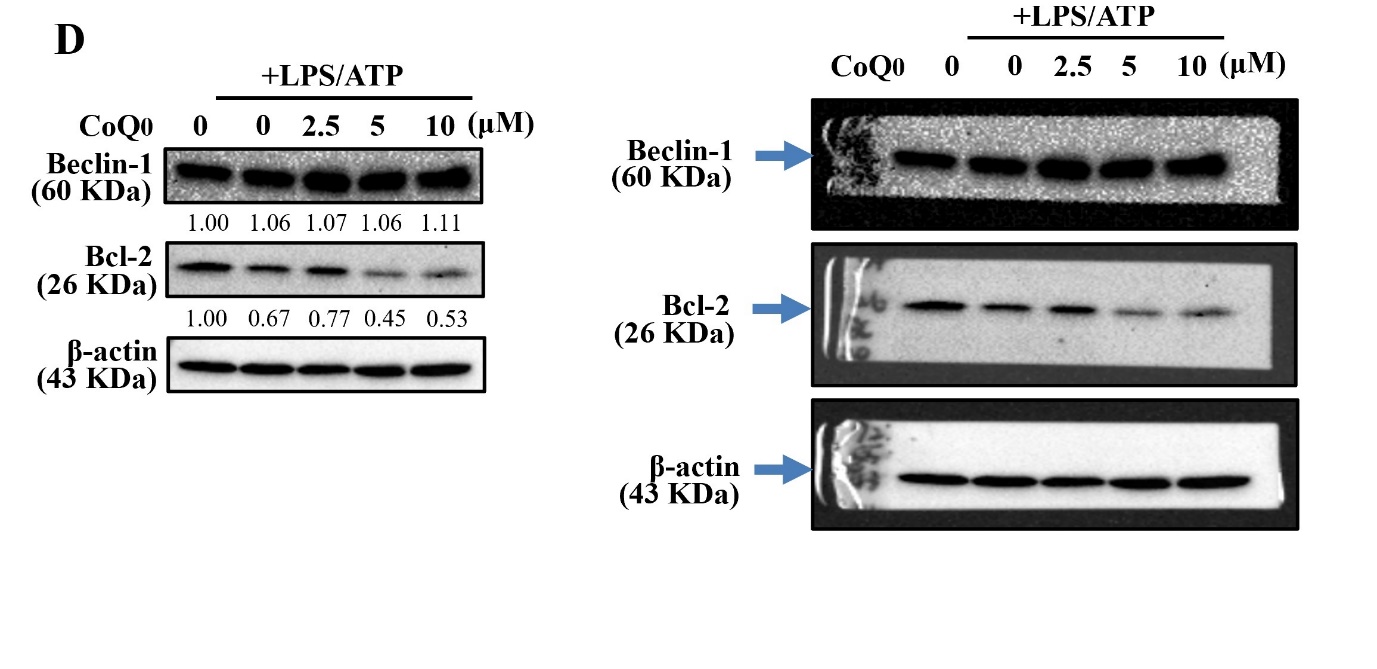


**Figure: S4A**


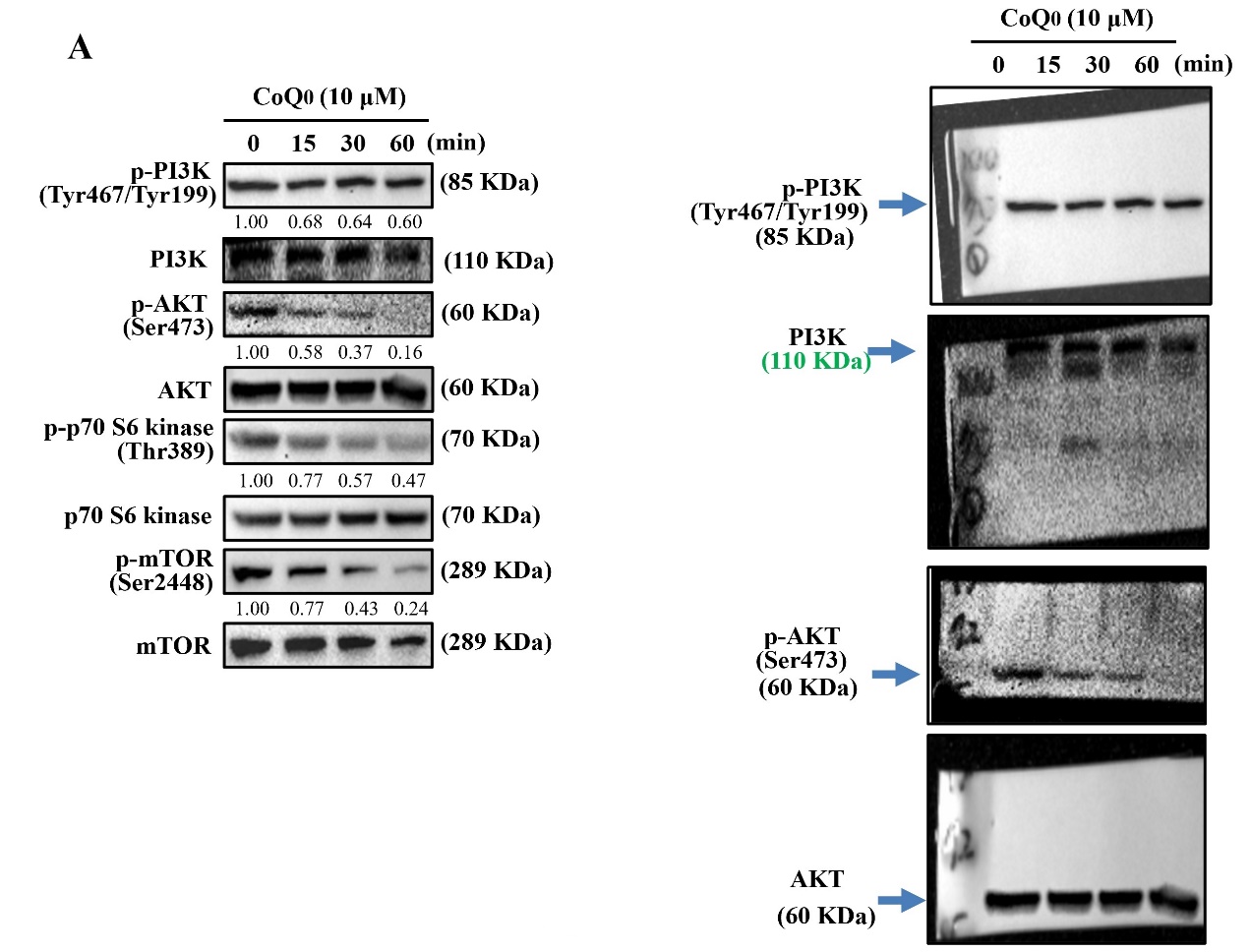


**Figure: S4B**


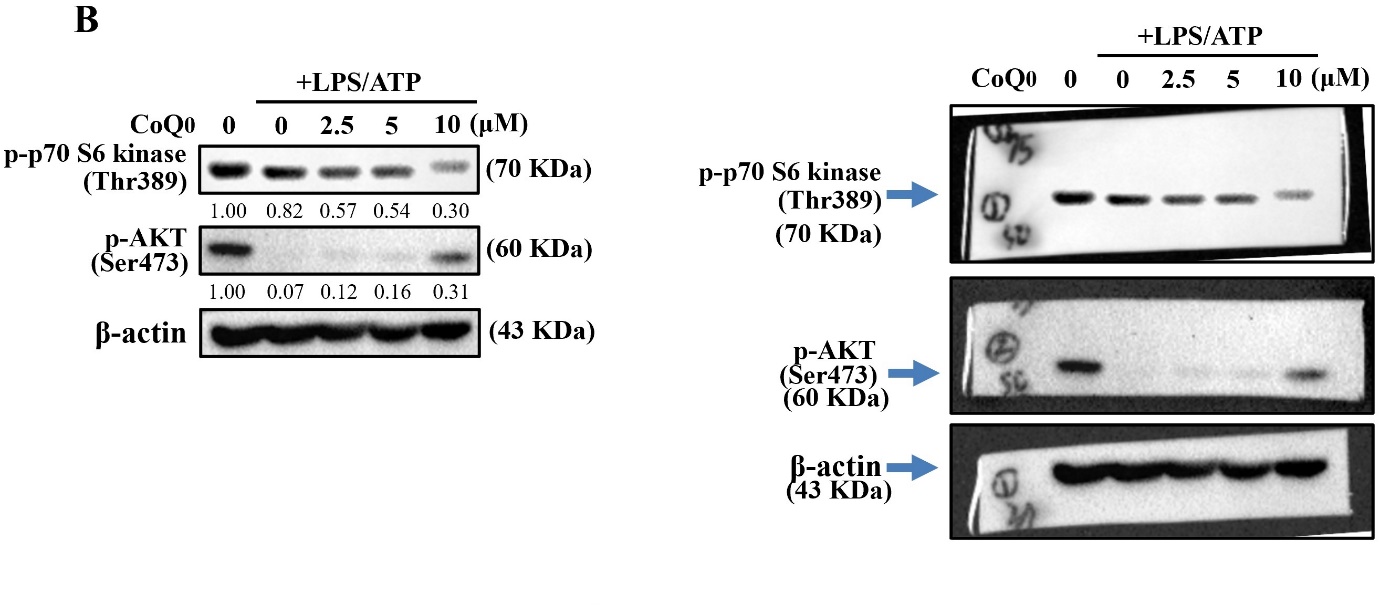


**Figure: S4C**


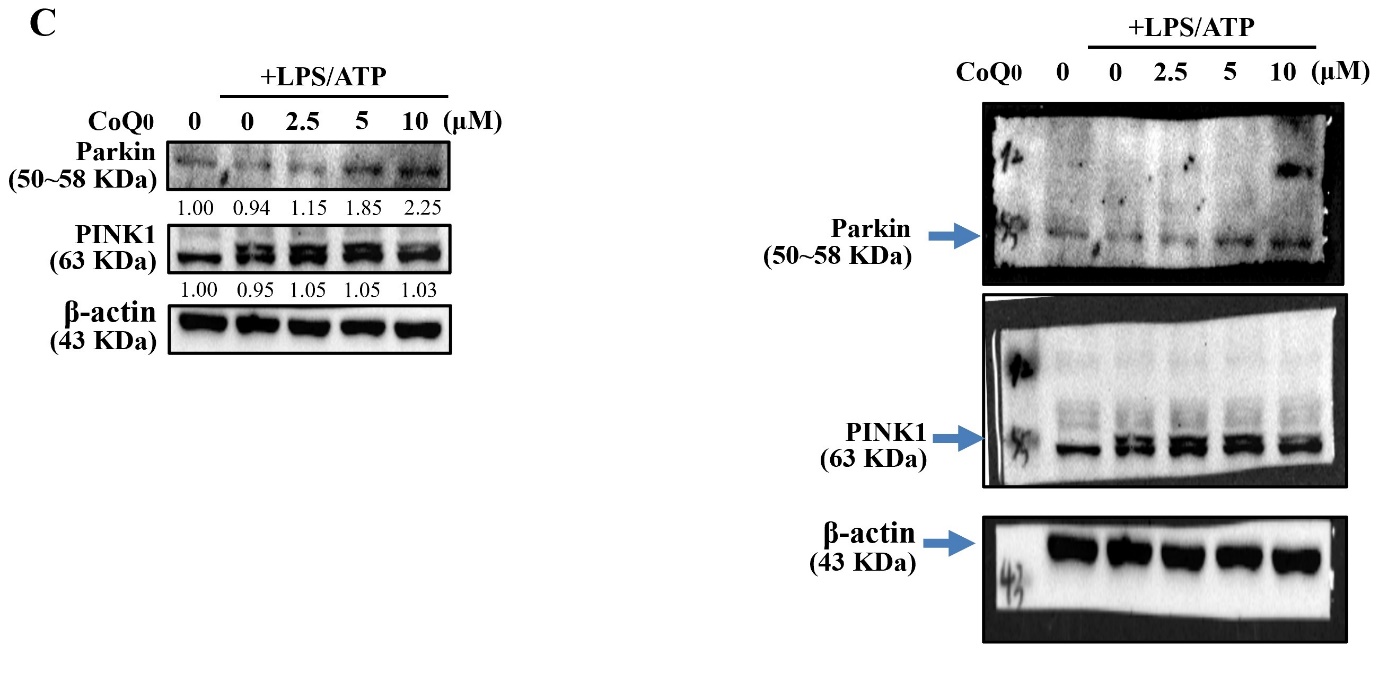


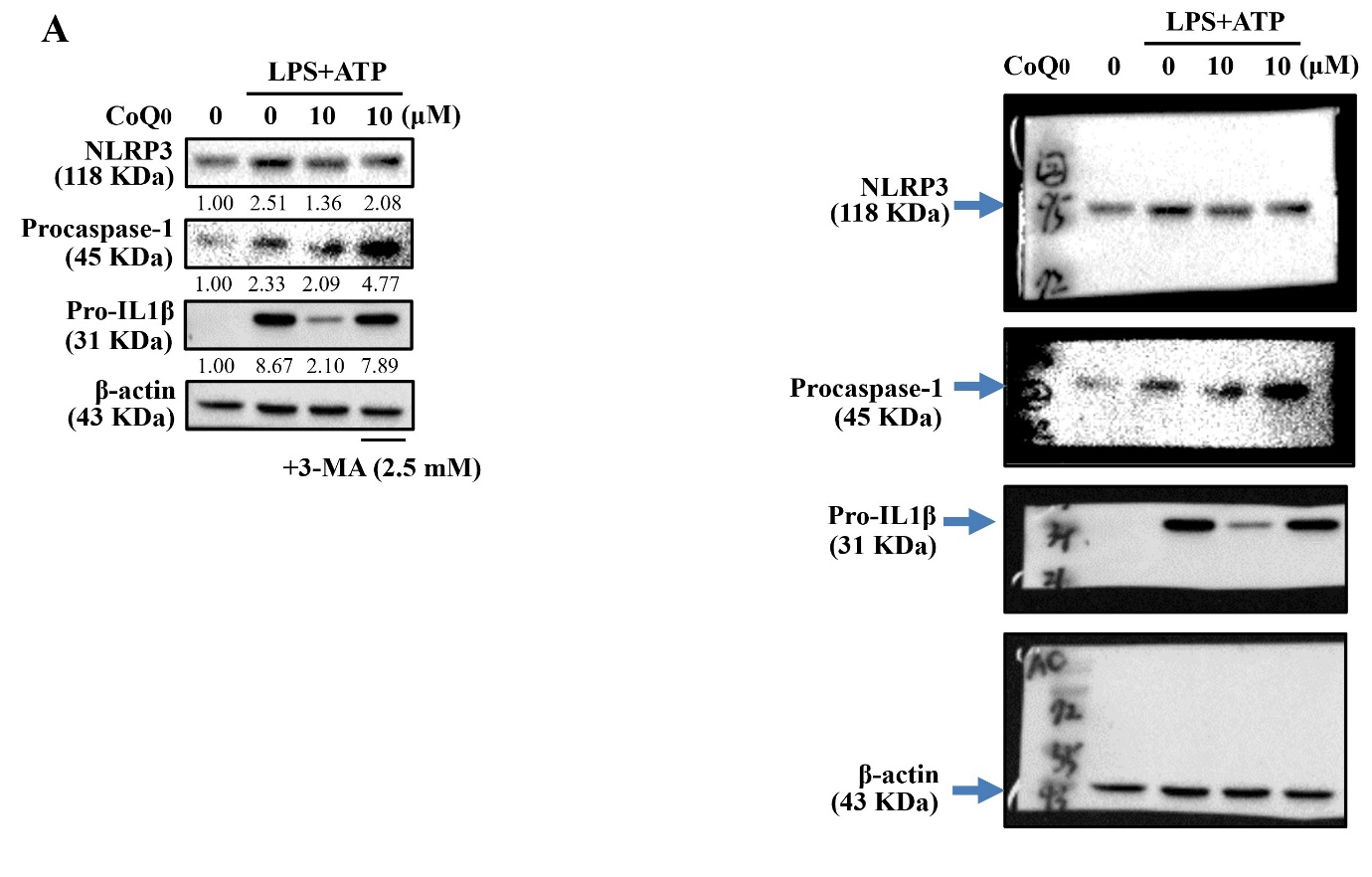
**Figure: S5A**

**Figure: S5B**


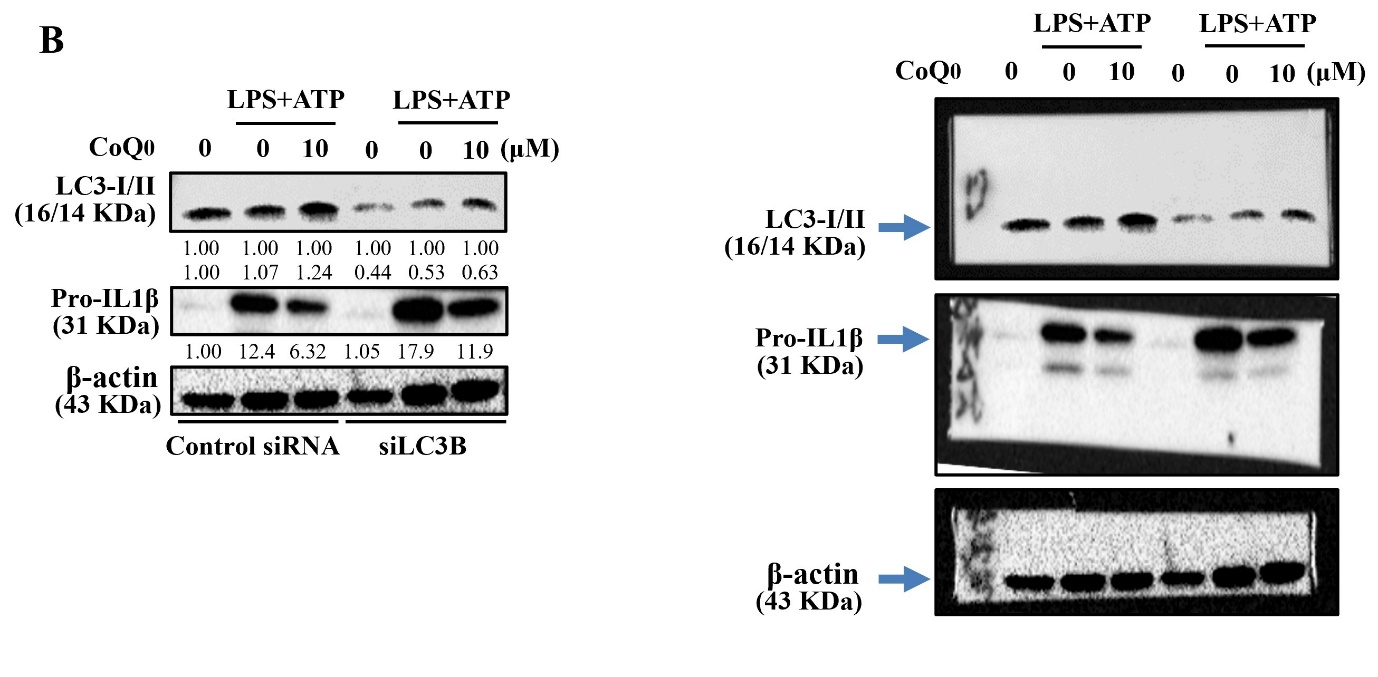


**Figure: S7B**


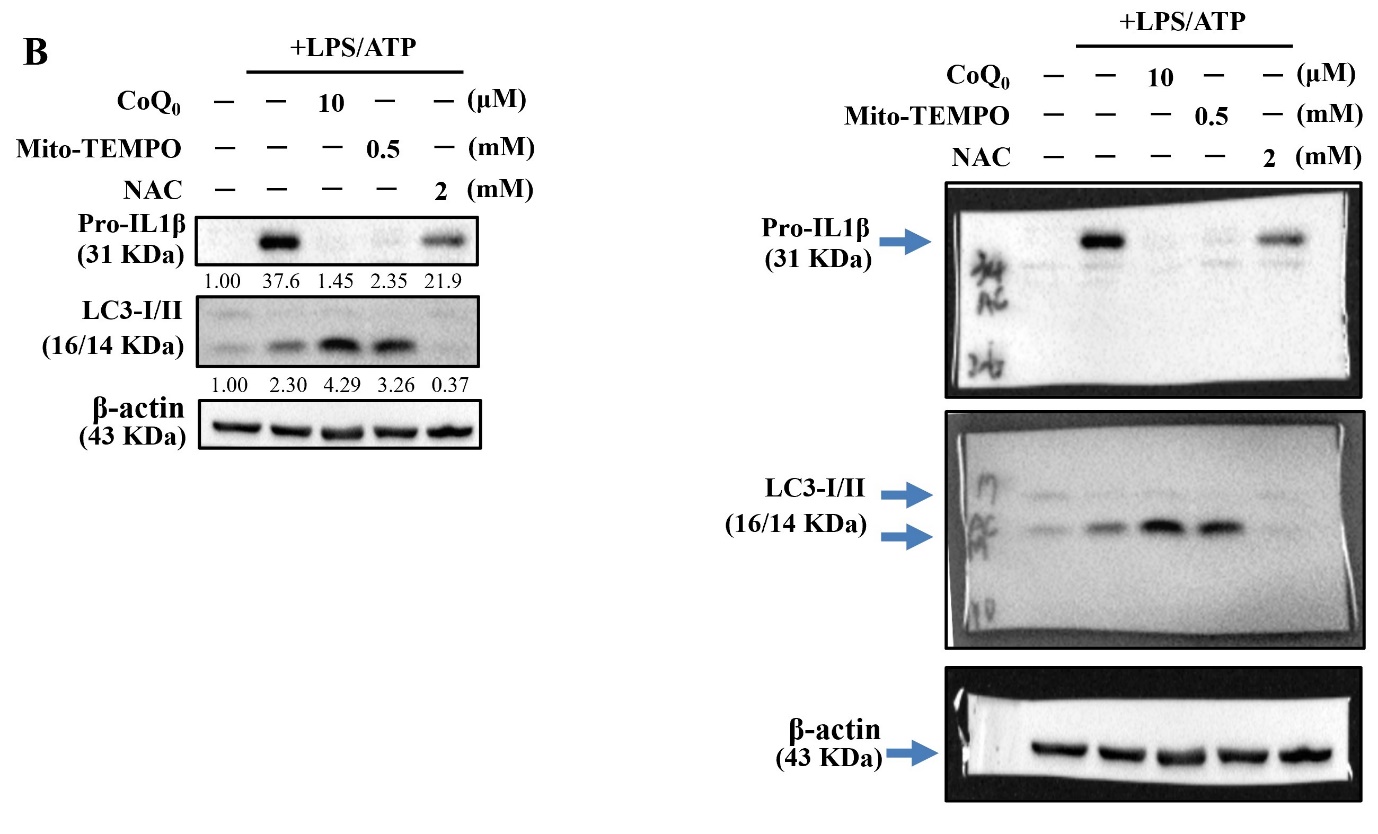


**Figure: S7C**
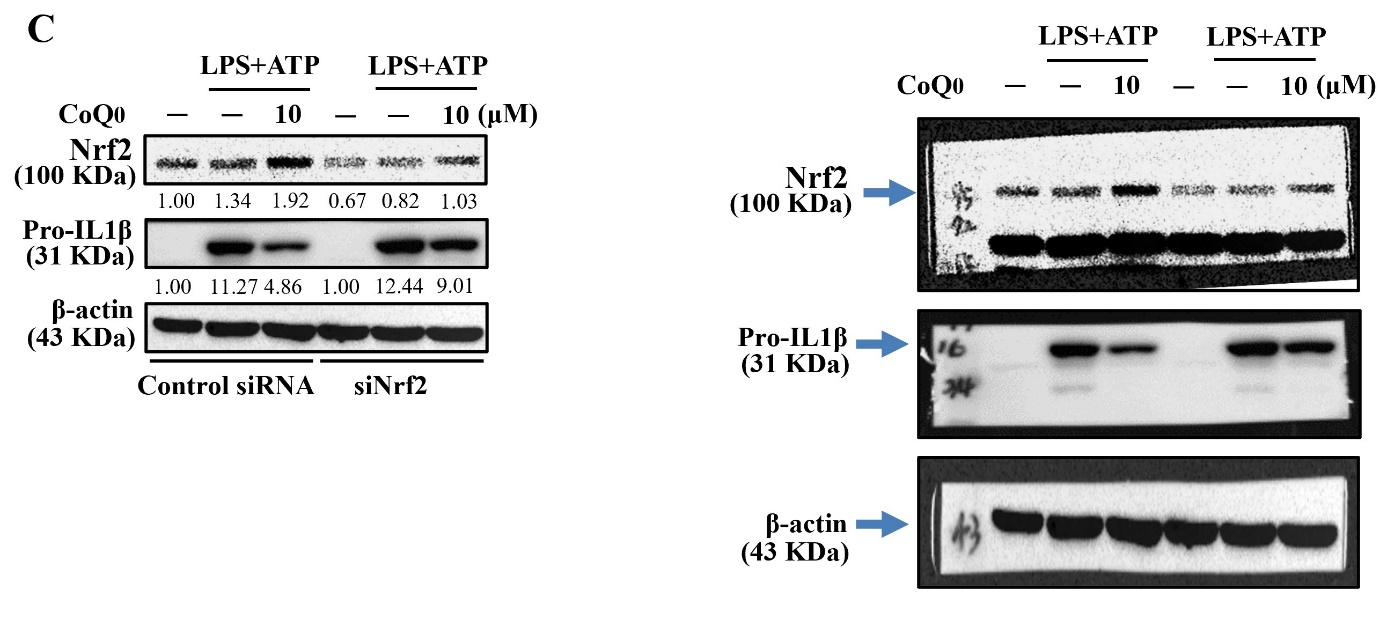

Supplement: Supplementary Materials — The file contains original raw images of Western blot figures in the manuscript. [file 4266214.f1.docx]
